# The focused quantitative EEG bio-marker in studying childhood atrophic encephalopathy

**DOI:** 10.1038/s41598-022-17062-w

**Published:** 2022-08-04

**Authors:** Sungura Richard, Shirima Gabriel, Spitsbergen John, Mpolya Emmanuel, Vianney John-Mary

**Affiliations:** 1 Department of Health and Biomedical Sciences, School of Life Science, Nelson Mandela-African Institution of Science and Technology, Arusha, Tanzania; 2 Department of Neuroscience, Western Michigan University, Kalamazoo, MI USA

**Keywords:** Neuroscience, Medical research, Neurology

## Abstract

Although it is a normal involution process in advanced age, brain atrophy—also termed atrophic encephalopathy—can also occur prematurely in childhood as a consequential effect of brain tissues injury through trauma or central nervous system infection, though in both normal and premature occurrences this condition always presents with loss of volume relative to the skull. A common tool for the functional study of brain activities is an electroencephalogram, but analyses of this have reportedly identified mismatches between qualitative and quantitative forms, particularly in the use of Delta-alpha ratio (DAR) indices, meaning that the values may be case dependent. The current study thus examines the value of Focused Occipital Beta-Alpha Ratio (FOBAR) as a modified biomarker for evaluating brain functional changes resulting from brain atrophy. This cross-sectional design study involves 260 patients under 18 years of age. Specifically, 207 patients with brain atrophy are compared with 53 control subjects with CT scan-proven normal brain volume. All the children underwent digital electroencephalography with brain mapping. Results show that alpha posterior dominant rhythm was present in 88 atrophic children and 44 controls. Beta as posterior dominant rhythm was present in an overwhelming 91.5% of atrophic subjects, with 0.009 p-values. The focused occipital Beta-alpha ratio correlated significantly with brain volume loss presented in diagonal brain fraction. The FOBAR and DAR values of the QEEG showed no significant correlation. This work concludes that QEEG cerebral dysfunctional studies may be etiologically and case dependent from the nature of the brain injury. Also, the focused Beta-alpha ratio of the QEEG is a prospective and potential biomarker of consideration in studying childhood atrophic encephalopathy.

## Introduction

As the main organ of the central nervous system, the brain is integral to all human activity yet this organ goes through much over a lifetime. Indeed, from its origin in neural tube formation the brain usually passes through different stages of development in intrauterine life^1^, further development occurs during adolescence and adulthood. In old age, ultimately experiences involution that manifests in loss of volume, which is also known as brain atrophy or atrophic encephalopathy^2^. In certain circumstances, however, such atrophic encephalopathy may occur prematurely in childhood as a response to brain injury from varying etiological causes, with the most common causes of such offending factors on the brain being central nervous system infection^3^, mechanical head trauma^4^, anoxic injury from deprivation of oxygen supply to the brain^5^, metabolic-related brain injury such as renal and liver failure^6^, but also space occupying lesions and hydrocephalus, among many other causes^7^. Therefore brain atrophy is multifactorial in origin.

In the initial stage, brain injury may evidence with cerebral edema in the form of interstitial or cytotoxic edema. This disturbs the blood brain barrier (BBB)^8^, which results in fluid extravasation into extracellular space or intracellular fluid shift to the neurons after the damage of cell membrane sodium pump^9^. An increase in neuronal intracellular fluid results and this phenomenon is known as cytotoxic edema^10^.

As fluid accumulation occurs in the acute inflammatory phase of brain injury, brain volume may increase and neuroimaging may demonstrate evidence effacement of sulcal spaces, Silvian fissures and ventricular size^11^. Functional diagnostic tools such as EEG may demonstrate different pathological wave patterns in terms of frequency and even amplitude change^12^. Among the common manifestations of the pathological qualitative EEG is the appearance of theta and delta waves in the frequency range between 5–7 Hz and 0.5–4 Hz respectively^13^.

Despite their altered pattern in brain pathology, the brain waves may occur in varying physiological modulations in which EEG synchrony and desynchrony may be exhibited based on neurofeedback trainings^14^. Further, the hemispheric dominance may also present with a patterned reduction in one side Alpha power which is also defined as Alpha desynchronization^15^. Most of the trainings are done through introduction of feedback of the brain’s own generated waves at particular cortical site in real time using EEG to modulate cognition and behavior^16^ in which Alpha waves are thought to improve memory in upper frequency^17^. The magnetic encephalography (MEG) has been used in one study to enhance posterior lateralization of the Alpha rhythms^18^. Another utilized modality is the use of functional MRI (fMRI) through visual, auditory and tactile as selective attention tasks^19^. The EEG, MEG and fMRI with high temporal resolution are used to modify power of brain oscillation in different frequency bands involving Alpha, Theta, Beta and Gamma^20^. The most common among these waves is alpha-neurofeedback which is a frequent practice as a novel therapy that trains individuals to volitionally increase their alpha power for pain relief^21^. High alpha powers are commonly recorded in the parietal lobes as they are responsible for special role on attention^14^. In addition, Alpha waves are affected by visual perceptual pathways^22^. Further studies have also shown that parietal lobes are the prominent areas of Alpha synchrony^23^. In a relaxed state with eyes closed the brain generates uniform alpha waves in both hemispheres known as Alpha synchrony^24^. When there is a presence of coherent Alpha activities across large areas of the temporal cortex suggests that Alpha oscillations are synchronized between cortical regions. This is known as Alpha coherence^25^. Alpha block occurs when the Alpha waves are interfered by Beta activities are a result of external stimuli from the environment^26^. These dynamics in brain rhythms are fundamental parameters in neurofeedback science.

Another neurofeedback protocol is Alpha-Theta training which aims to achieve balance and maintain the ideal amplification and frequency of such brain waves through deep meditative and hypnotic like state^27^. It is also utilized in management of children with attention deficit hyperkinetic disorder (ADHD)^28^.

As long as this study was centered in pediatric age groups which bear challenges in some procedures in neurofeedback science^29^, it did not embark itself in training of special activities such as deep meditation to achieve neurofeedback practices for the sake of examining synchronized and desynchronized patterns of brain waves. Children were researched in a sober relaxed state to observe brain rhythms as natural as could possibly be with the summation of the total global values of numerical powers of Beta and Alpha rhythms to get the ratios in total values. In the current study the QEEG wave’s analyses were taken with eyes-closed state in which the Alpha waves are known to be prominent that they can be well measured against Beta waves^30^. This was done so to maintain resting state and prevent external stimuli that would modulate other brain’s wave’s patterns^31^. While most of brain rhythms studies on EEG neurofeedback are done for therapeutic purpose^32^, the QEEG power spectra in this study were dedicated for investigative role in a sober environment in order to explore the difference in outcomes between the normal childhood developed brains against that which has pre-senile atrophy.

Besides its natural development in senescence, brain atrophy is the long-term effect of brain injury^33^. With severe head injury, however, brain atrophy likely develops between 3 and 11 months after head trauma—a likelihood advancing according to severity of injury, especially when there is an associated loss of consciousness^34^. As neuroimaging is the modality of choice for studying anatomical brain changes through CT scans and MRI, EEG is among the most common tools dedicated to studying functional changes of the brain by depicting brain waves from the cortical layers.

EEG advancement came with the concept of quantitative EEG (QEEG), which also entails the application known as brain mapping in which Fourier’s transformation is adopted to give quantitative and pictorial image maps of the brain with different activity of wave power intensities with the goal of arriving into a more objective quantitative analysis of brain wave activities wherein the dipoles from the palisade layers may be located and presented in a topographic image view using special algorithms such as dipole localization methodology (DLM)^35^. QEEG thus uses the power intensity of different wave frequencies from different head regions to generate topographical images of numerical values of absolute or relative powers of alpha, beta, theta and delta wave frequencies^36^. These waves have hence allowed different indices to be developed, including Delta-Alpha Ratio, Theta-Beta Ratio and Beta-Alpha Ratio.

The Delta-Alpha Ratio is the most commonly utilized biomarker in studying cerebral dysfunction, especially when it is above 3.7 value^37^, perhaps because it can be case specific, especially in acute conditions like ischemic stroke^38^. Although literature that addresses QEEG in chronic brain conditions such as chronic stroke is sparse^39^, according to Sungura et al. (article in press) the Delta- Alpha Ratios in extant literature have shown insignificant correlation with atrophic encephalopathy being a chronic condition with which those affected present with convulsive disorders, loss of memory or tremors. Further comparative studies show that the QEEG’s Delta-Alpha Ratio is higher even in sub-acute stage of stroke and increases with the size of stroke as measured by the National Institutes of Health Stroke Scale (NIHSS)^40^. A significance value of relative powers of Alpha, Theta, Delta, Delta-alpha Ratio, Delta-Theta/Alpha–Beta Ratio has also been suggested in post -stroke evaluation of brain injury in animal studies^41^.

As most cases of brain atrophy in children present with overwhelming beta activities in resting condition^42^, this study examines the relation of the Beta-Alpha Ratio in atrophic encephalopathy by focusing on occipital electrodes as the reference point of the head region hence designated this QEEG indices as Focused Occipital Beta-alpha ratio (FOBAR). Excess beta activities have also been reported in children with Attention Deficit Hyperactivity Disorder (ADHD)^43^, so the overwhelming Beta activities in children with atrophy encephalopathy must be given gravity of further studies.

The rationale for FOBAR is that, in a normal EEG at rest, alpha rhythm tends to dominate the posterior head region and hence this is why it is almost always depicted with relatively high amplitude in the occipital electrodes (O1 and O2)^44^. The Beta rhythm tends to be dominant in the frontal head region and may be concurrently interfered with discharges from the eye blink artefacts^45^. Rarely, beta rhythm replaces alpha rhythm in a transitional state such as drowsiness, where alpha dropout is noted^46^. In ideal situations, the replacement of Alpha rhythm by Beta waves in a fully resting subject may be a marker of abnormality. This study therefore takes advantage of the Alpha disturbance in posterior electrodes to examine the newly proposed technique in QEEG.

This cross-sectional study involves children from Northern Tanzania who presented with brain CT scans from March 2013 to December 2020.

## Materials and methods

This study in Northern Tanzanian health facilities, specifically in sites that have CT scans installed for easy assessment of brain volume status, was conducted between January 2019 and December 2020 using a cross-sectional design.

### Study population and sample size

The current research involves a total of 260 children from Northern Tanzania under 18 years old who presented at radiology facilities for head CT scan examinations. It utilizes Fisher et al.’s^47^ statistical formula to compute the sample size at the confidence interval of 95% with a margin error of 5% and a prevalence of brain atrophy in children of 16.04%^48^. It intentionally uses Sungura et al.’s (article in press) population to follow a different approach of their brain function in the form of quantifying EEG wave patterns to study their relationship with brain atrophy.

All this study’s methods for CT and EEG observed the national and known international guidelines regarding standard operating procedures and regulations for ensuring quality and authenticity in this research, including its outcomes. Specifically, they followed radiological baseline of CT imaging and the 10–20 electrodes placement for EEG. Furthermore, the EEG acquisition and analysis were performed at a paper speed of 30 mm/s, a low filter of 1 Hz, and a high filter of 70 Hz. The ECG electrode was connected to aid in the identification of artifacts from cortical wave activities. We looked at EEG epochs that were 10–20 s long and were free of artifacts in accordance to standard guidelines and regulations in performing these procedures in medical fields.

**Figure 1:**
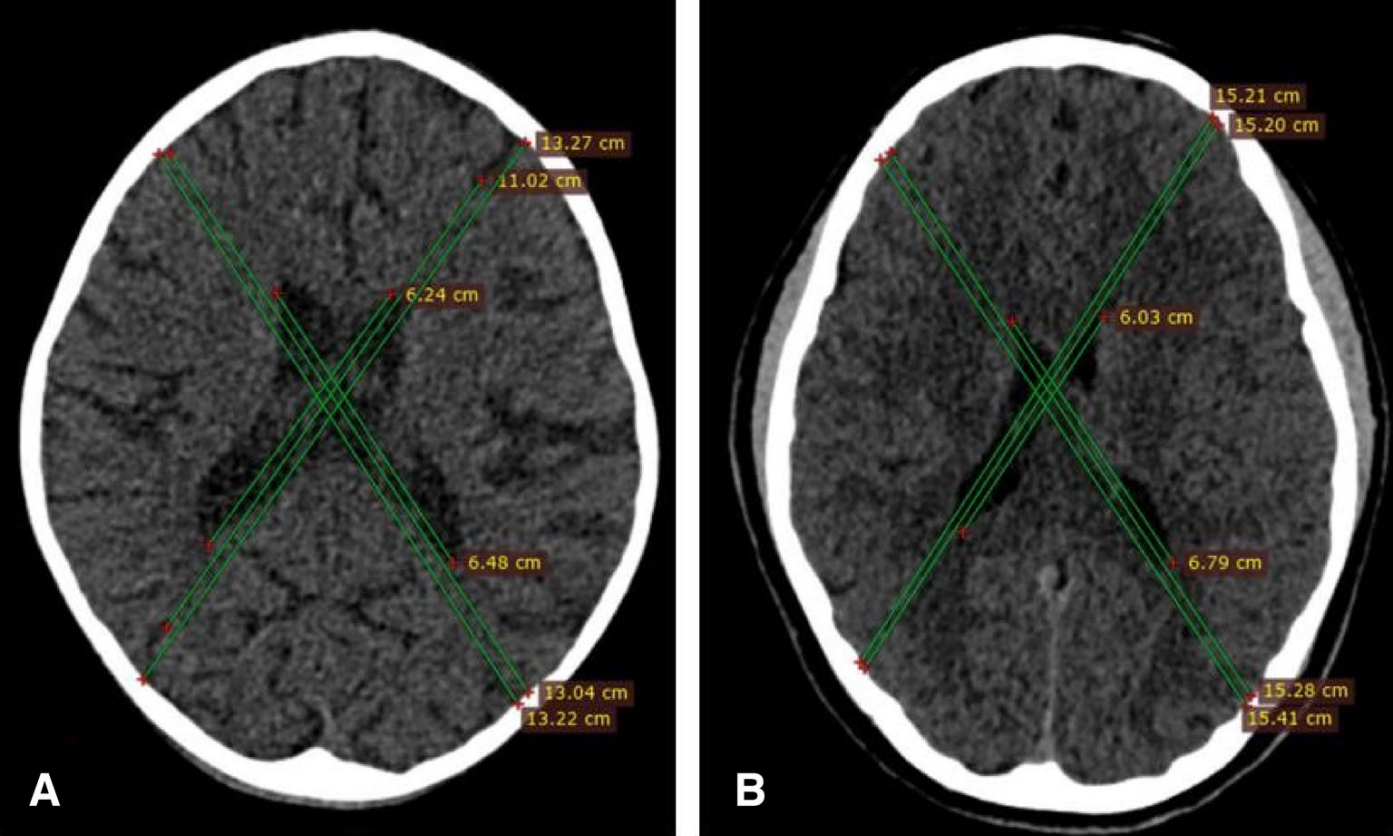
Brain CT scan sections at the widest part of the lateral ventricular body. (**A**) A case of brain atrophy from a 6-year-old male child showing prominent sulci and enlarged lateral ventricular body with a DBF value of 0.58 consistent with grade-II brain atrophy. (**B**) A normal brain volume of a 13-year-old male child showing effaced sulci intimately related to the calvarium, normal size lateral ventricular body with a DBF value of 0.81.

The participating children’s CT scans were examined to differentiate normal and brain atrophy cases using the diagonal brain fraction formula published by Sungura et al.^49^ (Fig. 1). In this, children who score a DBF of more than 0.75 are considered normal subjects, while those scoring 0.75 and below are deemed cases of brain atrophy.

### Matching cases and the control group

The matching of case and control groups in this research resembled that by Sungura et al. (article in press) as it was also done by age and gender but the current work had a ratio of 4:1 given its limited number of controls.

### Inclusion and exclusion criteria

Both atrophic cases and control subjects were enrolled after meeting the criteria of being less than 18 years of age, having had a CT brain scan and residing within Northern Tanzania but also passing exclusion criteria. Subjects were excluded by being outside the age or residence criteria but also if they evidenced space occupying lesions in the brain before surgery as this criterion maximized the utility of the DBF formula that follows anatomical landmarks.

### Procedures of the study

A 20-min EEG acquisition procedure was conducted while each subject was in a wakeful and resting condition using 10–20 international electrode placement systems and EEG recordings with digital manipulations involving Bi-polar longitudinal montage with eye closure and eye opening as well as Common reference montage using auricular electrodes A1 and A2 to generate the QEEG values analysis^50^. Further, the EEG recordings were done at the paper speed of 30 mm/s, low filter of 1 Hz and high filter at 70 Hz.

### Collected data

**Figure 2:**
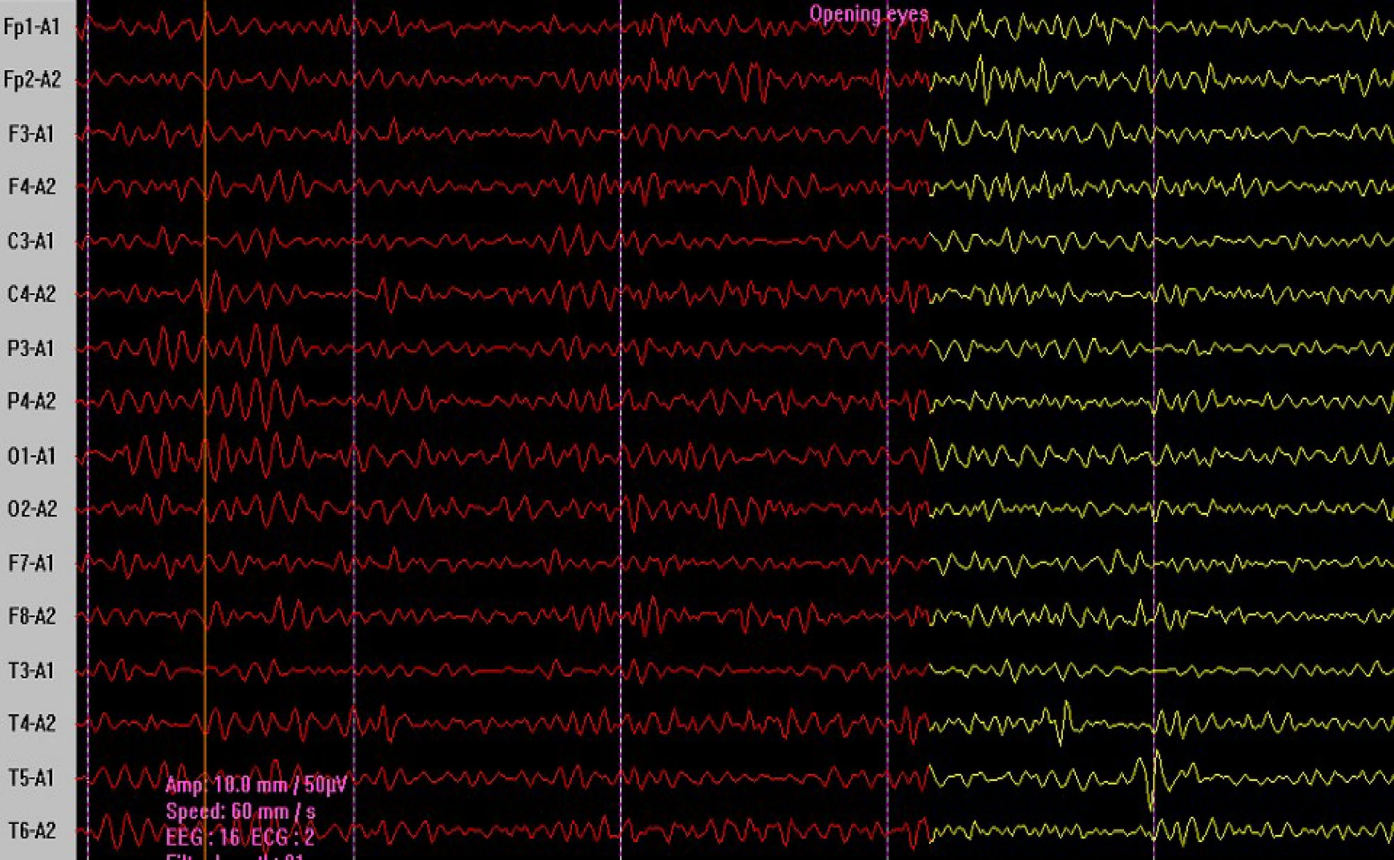
Normal EEG wave pattern. Normal alpha rhythm dominant at O1-A1 and O2-A2 posterior electrodes showing attenuation on eye opening in a pediatric subject. The waves recording show 10–11 ripples (Hz), implying brain cortical waves in alpha bad frequency. Amplitude suppression (yellow) after the opening of eye evidences reactivity that implies normal alpha waves. The posterior dominance is marked by prominent wave amplitude in the posterior electrodes (O1 and O2).

The collected data concerned brain volume in the form of DBF as described by Sungura et al.^49^, records of the dominant rhythm in occipital electrodes based on the known frequency range Alpha (8–12 Hz) (Fig. 2), Beta (13–30), Theta (5–7 Hz) and Delta (0.1–4 Hz) for qualitative EEG^51^.

**Figure 3:**
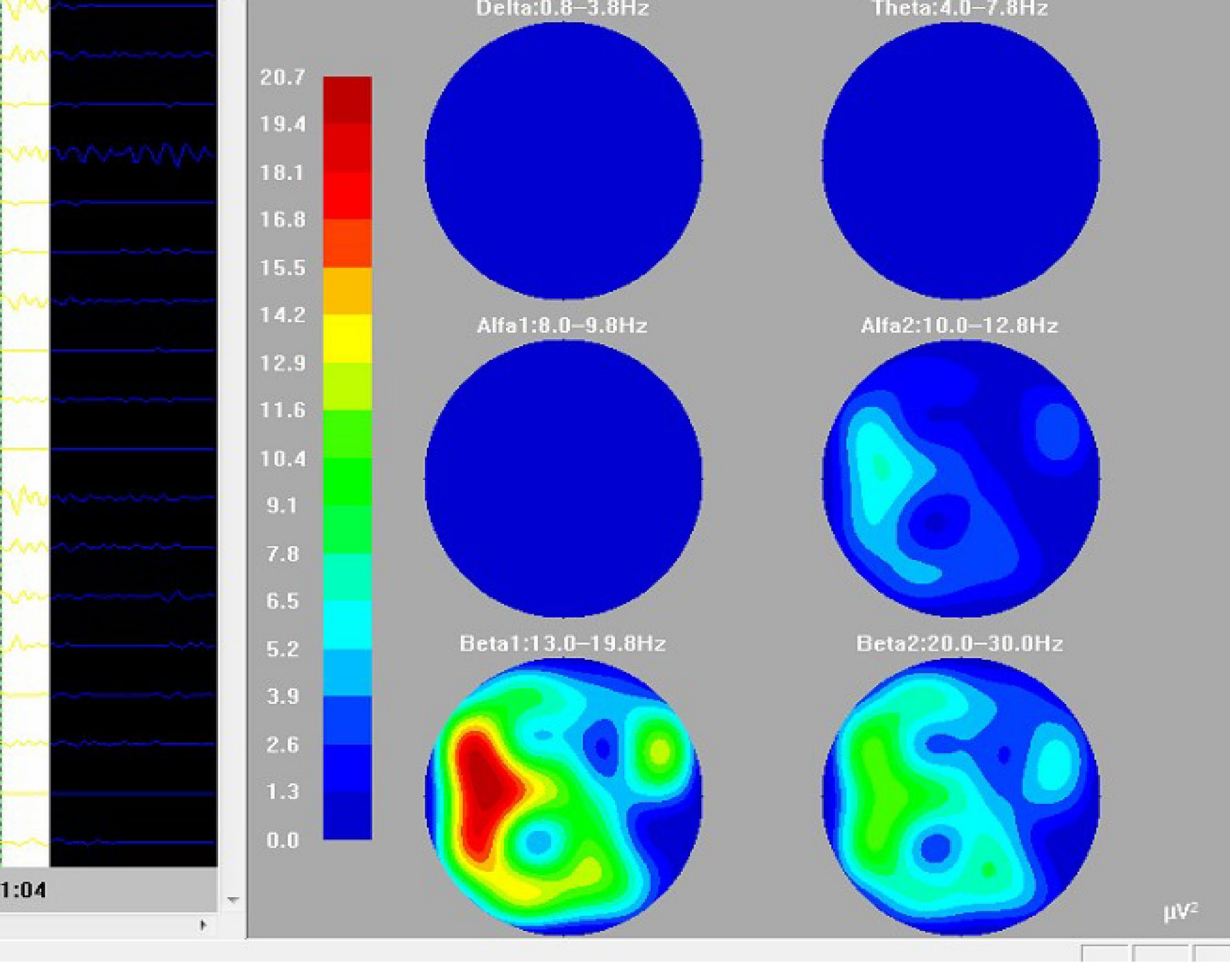
Brain colour spectral mapping of EEG power waves. EEG brain mapping showing signals in all frequency ranges with Delta and Theta being very minimal and Beta signals being the dominant rhythm in a male child.

**Figure 4:**
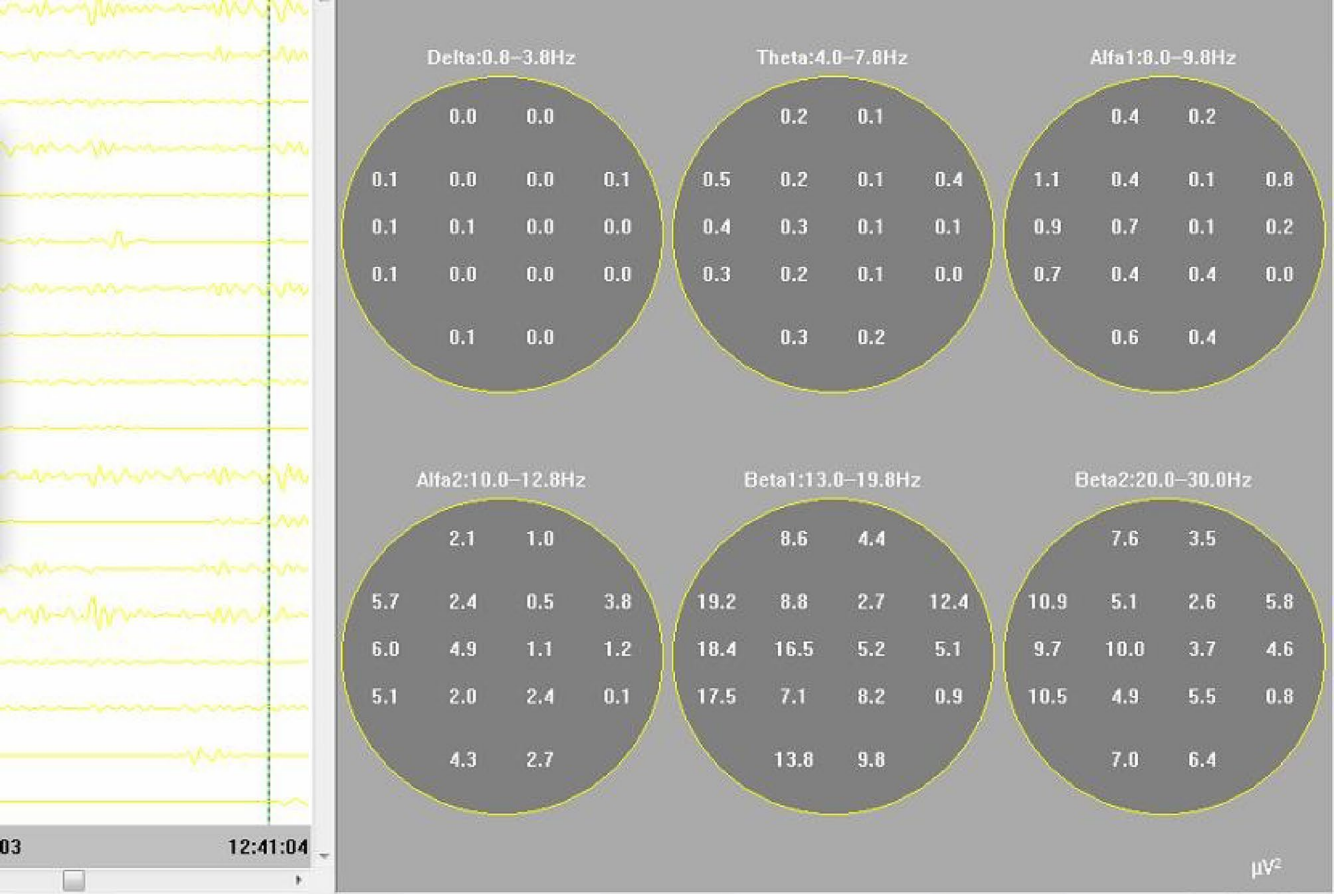
Numerical power intensity of the EEG wave form.

Furthermore, the QEEG values were analysed and recorded using Delta-Alpha ratio (DAR) (Fig. 3). The ratios were obtained by measuring the total numerical relative powers of the Delta band and dividing this by the Alpha power band obtained on the topographic spectrum^52^ (Fig. 4). This spectrum involves what is also known as brain mapping, which is recorded through Fourier’s transformation for which a DAR above the value of 3.7 is reported to be used as threshold for cerebral dysfunction^37^.

The quantitative EEG (QEEG) analysis included determining the Delta-Alpha ratio (DAR) and Beta-Alpha ratio (BAR) by calculating the total power of the Delta and Beta bands over the Alpha power band, as obtained from the topographic power spectrum recorded using Fourier's transformation. To achieve the above mathematical calculations, the transformation was performed using the EEG machine's built-in tool box, and actual manual counting of the numerical values for each electrode was performed.

Individual frequency bands were mapped in each topogram as Delta, Theta, Alpha-1, Alpha-2, Beta-1 and Beta-2 in varying color intensities. The degree of intensity of each frequency band was compared with the color spectrum with progressive increase in energy values from blue to red values. In the above patient, the highest power spectrum (red) is noted in the Beta-1 range in the left head region. The lowest power spectrum (blue) is noted in Delta, Theta and Alpha-1 frequency bands.

The BAR (beta-alpha-ratio) value was obtained by dividing the total values of occipital electrodes in beta by occipital the values in alpha ranges (13.8 + 9.8 + 7.0 + 6.4)/(4.3 + 2.7 + 0.6 + 0.4), giving BAR as 3.375. The numerical values of the power spectrum were obtained from the Fourier’s transformation of the EEG waves in all electrodes and presented in topograms according to the values of their power intensity and location in varying parts of the head region.

Statistical analysis was performed using R-program version 4.0.3 for computation^53^. In this program, descriptive statistics was done and presented in frequency (f) and percentage (%). Correlation was also performed to assess the linear relationship between DBF and Delta-alpha ratios as well as Beta-alpha ratios. Chi- square was used to assess association within categories of EEG parameters, and T-tests were used to test correlation within continuous EEG variables.

### Ethical consideration

The approval for conducting this research was obtained from KNCHREC ethical board on behalf of the National Institute of Medical Research (NIMR) to conduct the study in Northern Tanzania with a certificate number KNCHREC 0010. Written permissions to conduct this research were further obtained from the host facilities, and these covered aspects such as patients’ demographic data, archived CT scan images and laboratory results. Informed consent was obtained from all participants above 16 years of age. More specifically, individuals below 16 years who are also recognized as minors were involved through their parents who signed written informed consent form. CT scans were reviewed after meeting the standards of good diagnostic qualities. Also, participants under sober condition were subjected to EEG examination using 10–20 international electrode placement system. For this, wakeful EEG protocols were observed.

## Results

### Demographic characteristics of the study

**Figure 5:**
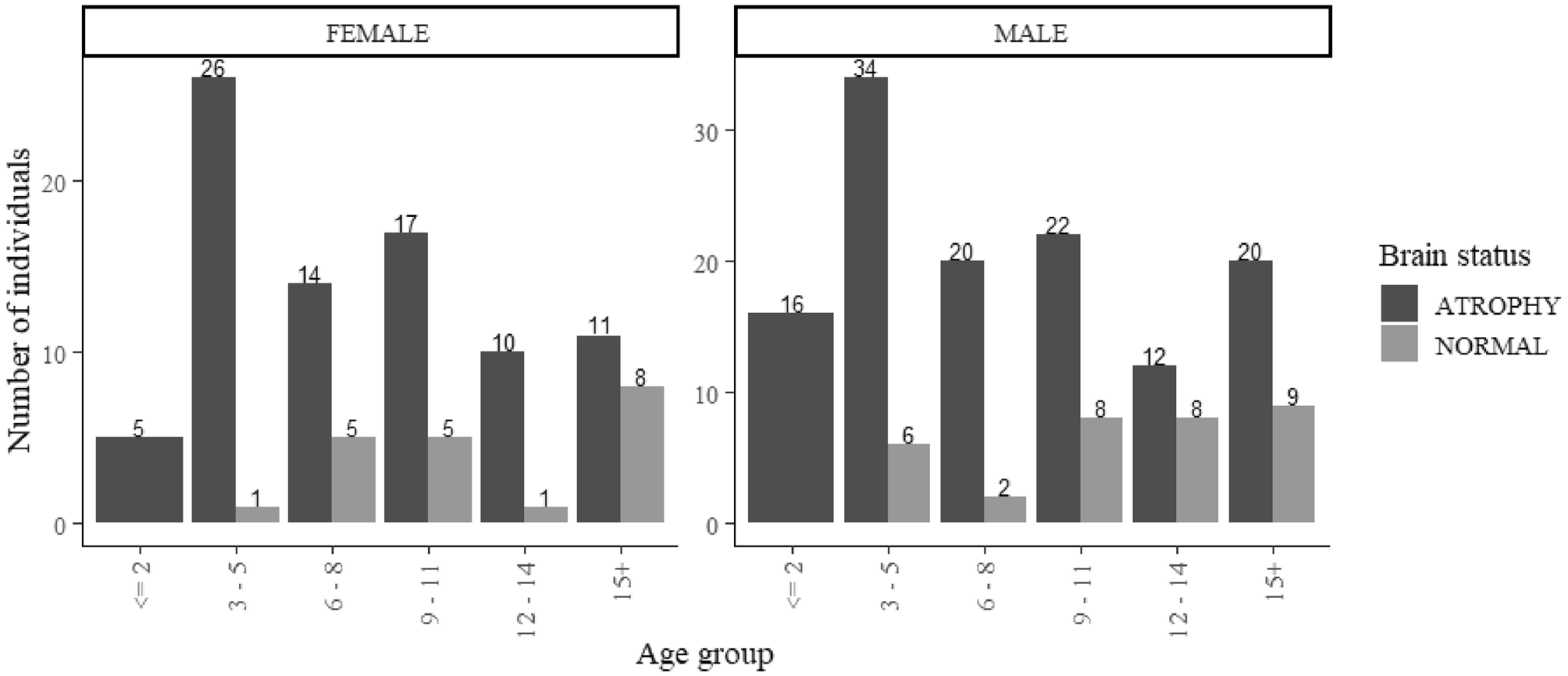
Participants’ age and gender demographics.

Of the study’s 260 total participants, 207 children had brain atrophy as evidenced by a CT scan and the remaining 53 children were normal subjects and thus formed the control group. The 157 males and 103 females were all under 18 years of age and fell into various age groups: 0–2, 3–5, 6–8, 9–11, 12–14 and 15+. Most atrophic cases were observed in the 3–5 age range for both female and male children, though this number is higher in male subjects. The high peak of the control group is in the 15+ age range. Figure 5 shows these and other specific findings.

Overall, these results indicate a trend of increased risk of brain atrophy in the very young age group and increased normality in higher age groups, with 9–11 years being the threshold point in age.

### Mismatching EEG parameters of children with brain atrophy

Although the results demonstrate three wave patterns in the form of alpha, beta and theta frequencies, a high propensity to generate Beta activities exists among cases of atrophic encephalopathy. Of the 106 children who exhibited Beta posterior dominant rhythm, 97 (91.5%) were atrophic cases and 9 (8.5%) were normal subjects. The increased Beta activities had statistical significance at the p-value of 0.009. The Alpha posterior dominant rhythm was shown in both cases and controls: of the 132 subjects who exhibited this wave pattern, 66.7% were cases of brain atrophy and 33.3% were control subjects. The Theta posterior dominant rhythm was rare and was observed in only 22 subjects with brain atrophy on a CT scan.

While Delta posterior dominant rhythm was not observed in any cases, the Beta frequency as the posterior dominant rhythm overrode the alpha and theta wave patterns. Regarding Delta-alpha ratios among cases of brain atrophy and their corresponding controls, no statistically significant difference was found. The DAR had a mean of 1.46 ± 1.73 among cases of brain atrophy and 1.62 ± 1.78 among the control subjects (see Table 1).

**Table 1:** Qualitative and quantitative EEG parameters.

Variables	Cases, n (%)	Controls, n (%)	Test	ODDS	*p* value
**PDR**			${\varvec{\chi}}$^**2**^ = 28.51***, df = 2		
Alpha	88 (66.7)		44(33.3)		
Beta	97 (91.5)	9 (8.5)		7.66	0.0091**
Theta	22 (100.0)	0.0		3.13	0.9967
**Delta alpha ratio**	1.46 ± 1.73	1.62 ± 1.78	t = − 0.59^ ns^, df = 79.14	1.38	0.1398
**Beta-alpha ratio**	7.53 ± 12.78	3.24 ± 4.18	t = 4.06, df = 244.69***	1.12	0.0605

Descriptive statistics n (%), means, chi-square test (x^2^, df), Two sampled t-test (t), Significance codes: '***'*p* < 0.001, '**'*p* < 0.01, '*'*p* < 0.05 ','*p* < 0.1

These results show weak relation between posterior dominant rhythm and Delta-alpha ratio and a fair relation between PDR and Beta-alpha ratio at a confidence level of 5%.

### Focused beta-alpha ratio as a specific QEEG biomarker

#### Focused beta-alpha ratio in brain atrophy

**Figure 6:**
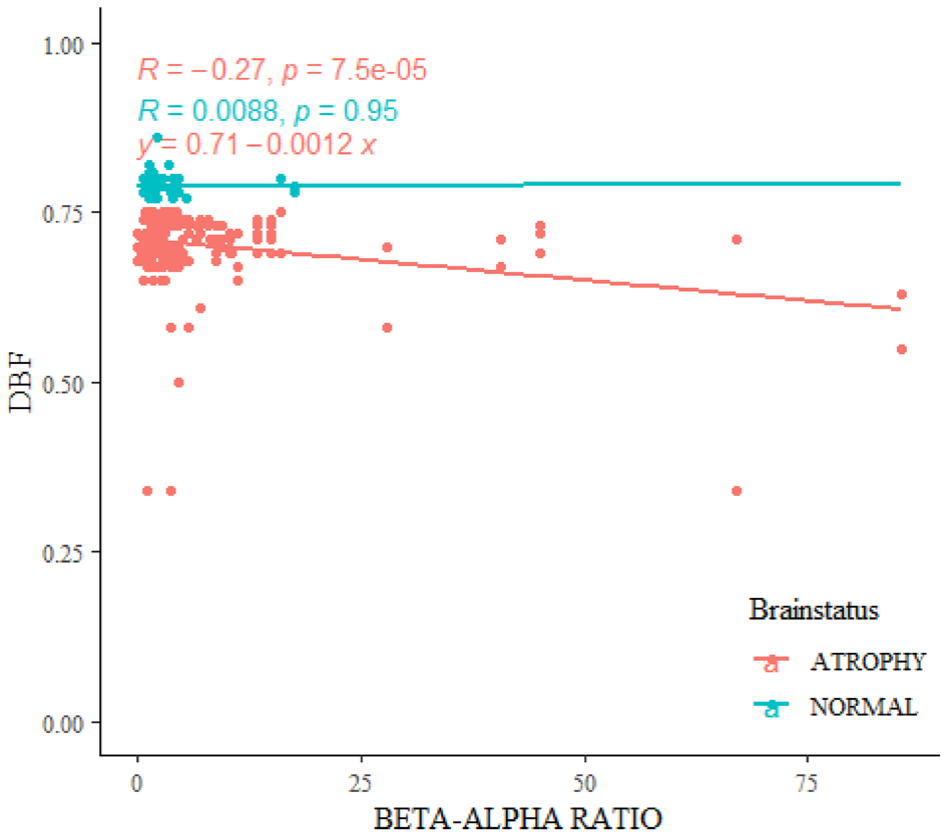
Beta-Alpha Ratio relation to brain volume.

The numerical values of Beta-Alpha Ratio, which were specifically taken in the occipital electrode channels, are key for the newly proposed biomarker known as Focused Occipital Beta-Alpha Ratio (FOBAR). The FOBAR shows significant negative correlation with brain volume in the form of diagonal brain fraction (DBF), with a *p* value of < 0.001 in the general study population. When group-wise analysis was done, atrophic cases still showed very high and statistically significant negative correlation between FOBAR and DBF. Interestingly, this negative correlation was less significant to normal subjects when independent analysis was performed in which a p-value of 0.95 was recorded among the controls (Fig. 6).

The focused occipital Beta-alpha ratio (FOBAR) showing negative correlation with brain volume with specific significance in cases of brain atrophy.

#### Gender consideration in relation between DBF and FOBAR

**Figure 7:**
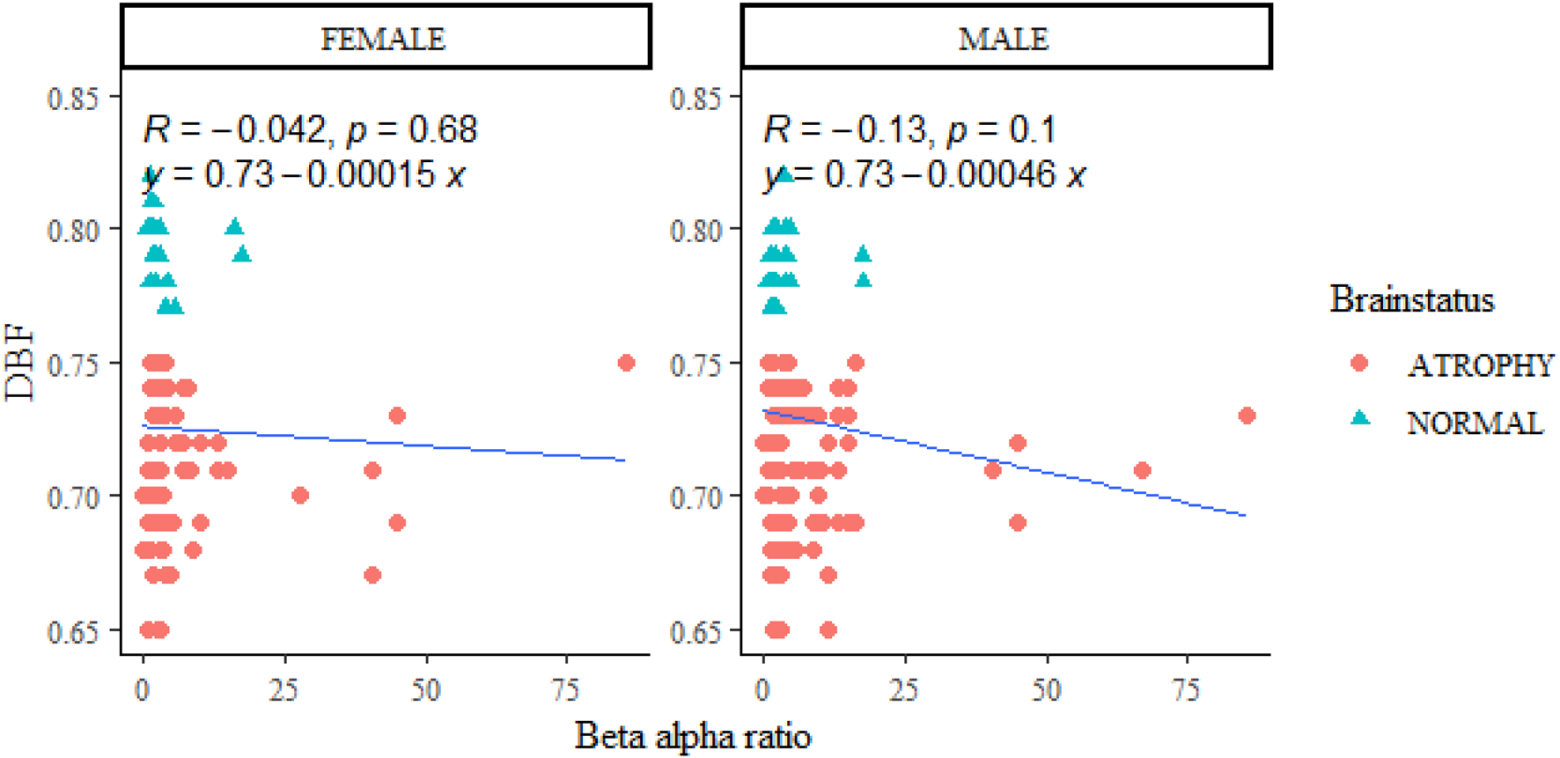
Gender relation of focused occipital bar. No significant difference between male and female subjects was found, despite an increased trend towards male children.

Despite the negative correlation in both gender differences, there is no significant statistical difference between female and male subjects with a p-value of 0.68 and 0.1 for female and male subjects respectively (Fig. 7).

#### Age distribution focused occipital Beta-alpha ratio in childhood

**Figure 8:**
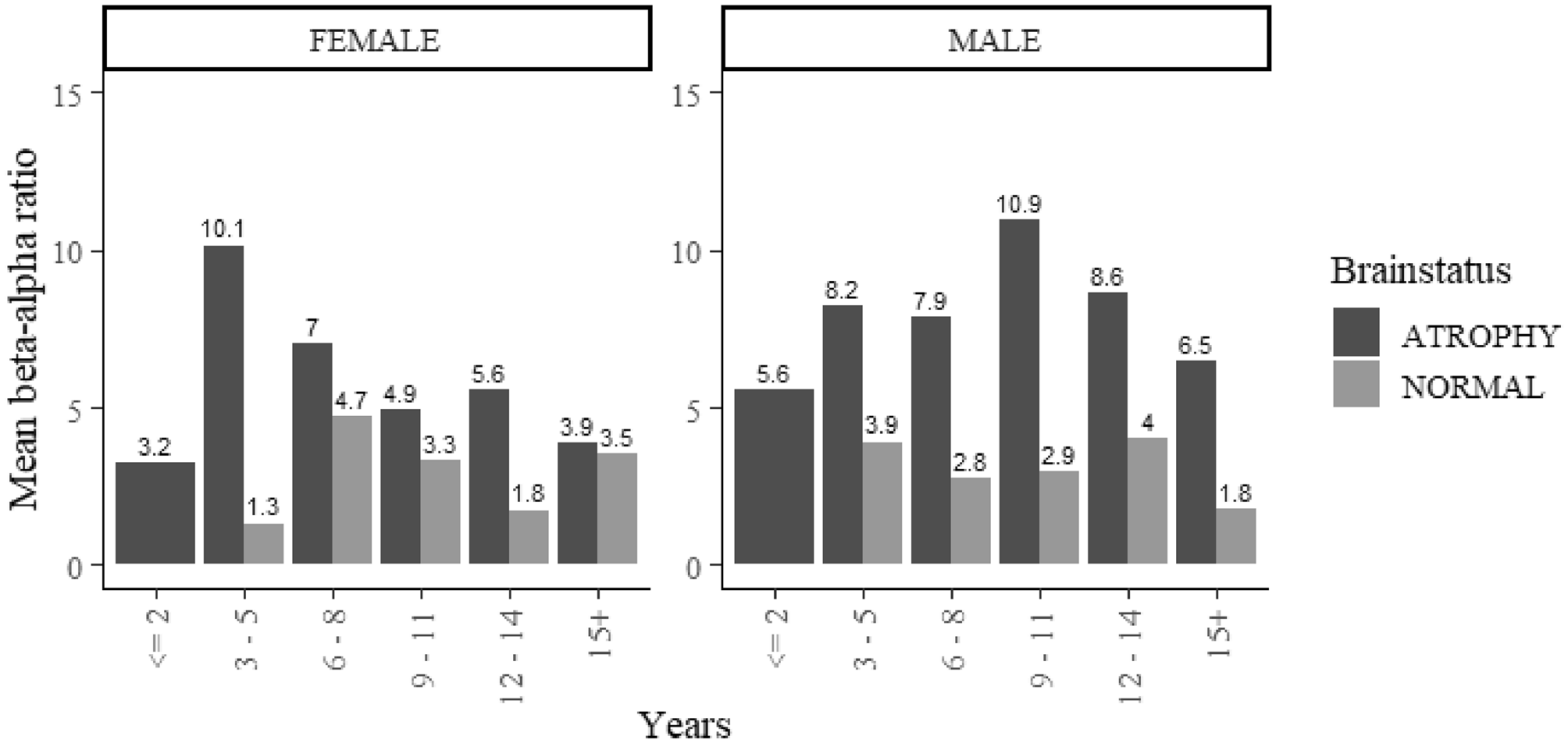
Varying trend of Focused Occipital BAR with gender. There is varying age in the peak values of the mean BAR with a constantly increasing trend in male children with brain atrophy.

The Mean focused occipital BAR presents with the highest peak at the age of 3–5 years among female children and 9–11 years among male children. The lowest Mean focused occipital BAR was observed at below 2 years of age in both male and female children. However, male children had slightly higher BAR values compared with females at the lowest age extreme and at 9–11 years among females (Fig. 8).

### Quantitative relation between Focused occipital BAR and DAR in brain atrophy

**Figure 9:**
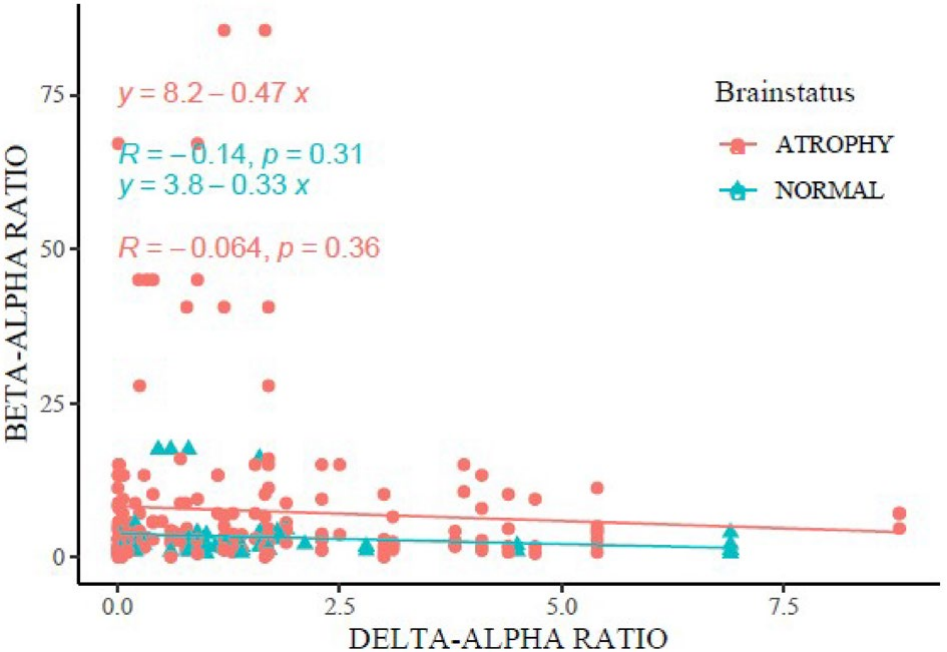
Correlation between Focused Occipital BAR versus DAR. In both atrophy and normal subjects, BAR shows insignificant correlation despite a trend towards children with normal brain volume.

The focused Beta-alpha ratios taken in occipital electrodes correlate negatively with the well- known Delta-alpha ratio in studying encephalopathy. This correlation is insignificant statistically both in atrophic cases and in normal subjects with the p-value of 0.36 and 0.31 in atrophic and normal subjects respectively (Fig. 9). Further analysis shows insignificance also when gender variation is considered, although a slight trend of significance leans more towards male children (data not shown).

## Discussion

In this and other studies, male children were generally availed enormously in the service of head CT scans^54^, suggesting an increasing rate of hospitalization and utilization of computed tomography among male children compared with their female counterparts^55^. This can be attributed to behavioral differences, whereby male children tend to be associated more with traumatic events that require neuroimaging services such as a CT scan and MRI for gross visualization of brain architecture. It is observation shared by Bellolio et al., who deemed the male gender an important predictor of increased CT scan usage in emergency departments^56^, and by Mettler et al. given their result concerning how more male than female children had used the hospital facility of head CT scan investigation^57^.

Regarding age, the current study finds an increasing trend of neuroimaging with control subjects in which the peak incidence was noted at the age of above 15 involving children who were not cases of brain atrophy. An important finding in this work is that the peak age of brain atrophy in the studied population is 3–5 years for both male and female children. To the best of our knowledge, pediatric brain atrophy has been scantly studied in a larger sample size than the current study. A fairly large study in Cameroon using children’s CT scans revealed about 28.5% of children with epileptic convulsions having brain atrophy and that most had convulsions at the age of above 2 years, as reported by Moifo et al.^58^. As the risk of brain atrophy in childhood has been found to be higher with those under the age of 5 years^48^, the implication is that 2–5 is the riskiest age range for brain atrophy in childhood, though there are variables to consider here. While the former considered convulsions the latter was concerned with risk relating to CNS infections such as meningitis, cerebral malaria and HIV encephalopathy, and this is besides population/demographic variations so it is not so straightforward. In this work, the authors present the childhood atrophy experience found in Northern Tanzania.

The current study observed an increasing trend of beta activities in children with brain atrophy regarding posterior dominant rhythms. Some children retained the normal alpha posterior dominant rhythm in a resting state but without statistical significance. Overall, 91.7% of children with brain atrophy presented with overwhelming beta posterior dominant rhythm, though this observation was not matched with the Delta-alpha ratio (DAR)—the most common QEEG biomarker that is mostly used for studying cerebral dysfunctions^37^. This finding is possibly because of the sub-optimal generation of Delta activities in children with brain atrophy that this study observed. According to Tolonen et al., Delta activities tend to increase in the background of cerebral edema as a result of acute cerebral injury by means such as ischemic stroke and, the authors add, concurrent with this is diminution of Alpha activities^59^. Further studies have reported that Alpha activities represent good integrity of the synaptic network in the cortical part of the brain, hence representing synaptic damage of the neurons^60^. Therefore, inasmuch as Delta signals tend to be lower in the absence of edema, the DAR values stand to be trivial biomarkers for in-depth studying of cerebral dysfunction in the setting of brain atrophy. In the current study Beta activities are high among cases of brain atrophy, largely because of explosive fast epileptiform activities, failure to relax and use of Benzodiazepine drugs^61^. Also, increasing Beta activities may be because of thinning of the brain parenchyma from the atrophic process that result in accentuated expression of the fast waves due to reduced absorption coefficient^62^. An increased Beta expression in brain atrophy may also be the result of compromised Alpha generators due to pathological changes that result in lower Alpha bowers. In tandem with the study by Finnigan et al. (2007), the current work deems Beta activities to represent neuronal survival even though they may easily be overlapped by artifacts such as those with high frequency range^40^. The current study focused on Beta activities at the occipital head region to avoid other artifacts such as eye blinks and frontalis muscles, among others.

The lower significance of DAR could be attributed to low generation of slow waves in the cortical brain, including both theta and delta waves. The theta waves were the only slow waves observed in this study and happened to be dominant in posterior electrodes in only 22 subjects with brain atrophy, while a comparable study by Zhang et al. reported tendencies of post-stroke increases in Theta activities^63^. This current study nevertheless did not confine itself to cerebral infarct alone, even though cerebral infarct may result in brain atrophy as a long-term consequence^64^. This work thus attributes the phenomenon of low DAR values to the fact that brain atrophy occurs in a chronic stage of tissue injury where more water content in the form of edema is drained out of the extracellular space where EEG waves are normally depicted^65^. Extracellular fluid being desicated increases transmission of the high frequency waves, simulating what is known as breech rhythm^66^. This is contrary to acute brain conditions like viral encephalitis, where brain tissue is inflamed and manifests interstitial edema wherein more water content is filling the extracellular space^67^. In support of this concept, Goloborodko et al. reported that it is edema in the terminal dendrites that is responsible for associated high Delta activities^68^. The cerebral edema causes thickening of brain volume that attenuates the high frequency cortical waves and flares slow frequency waves such as Theta and Delta^69^. Notwithstanding the fact that most studies of QEEG indices predominantly focus on strokes, Finnigan et al.’s work adds that DAR followed by Delta-Theta, Alpha–Beta ratio (DTABR) and the relative power of the Delta are the most important classifiers of acute ischemic stroke^70^. It is presumed that in such conditions, or in cases of brain tumor, the DAR as a biomarker for cerebral dysfunction gains its importance.

As the current study demonstrates limitation of the DAR indices as a biomarker for studying atrophic encephalopathy but also a significant trend of beta activities yielding in cases of brain atrophy, a focused Beta-alpha ratio biomarker was designed to target the occipital electrodes (O1 and O2), which are known to be the reference points for studying background activities of the brain^52^. In normal condition and in a resting state, these electrodes are dominated with Alpha waves as the posterior dominant rhythm, so this work assumes that replacing Alpha dominance represent abnormality. The analysis shows how this work’s Focused Occipital Beta-Alpha Ratio (FOBAR) gave a significant negative correlation with brain volume in the form of diagonal brain fraction (DBF)^49^ in the overall study population. Although studies such as Navea et al.’s have reported dominance of Beta activities in the frontal head region and hence differential Beta-Alpha ratio values^71^, to the best of the current authors’ knowledge no study has retrieved and found to have addressed the correlation between Beta-Alpha ratios in the settings of brain volume loss with purposive bias to the occipital electrodes. The outcome suggests there are more Beta activities than slow waves when a brain atrophies, adding more gravity in regarding the potential use of these bio- electric abnormalities in studying cerebral dysfunction relating to brain atrophy.

Regarding gender, this work found no significant statistical difference between female and male subjects, so the newly designed biomarker can be used independently despite some studies suggesting that male children have less Theta and more Alpha activity^72^. Therefore, age and sex may have no useful difference in the utility of FOBAR. The age distribution of the focused mean BAR showed high peaks at the age of 3–5 years among female children and 9–11 years among male children, which supports the idea that gender variation, derives from two related pathological processes. Specifically, some studies have shown that female children below 5 years of age are more prone to brain atrophy relating to infections such as urinary tract infections^67^, while male children at an older age are associated with traumatic events^73^. Also, studies have shown abnormal epileptic peaks at 8–11 years old^74^, which may confound the current study’s observation of male children peaking in terms of mean BAR values at 9–11 years old, since most epileptic discharges run in Beta frequency ranges. The Beta activities among others, may present in the form of polyspike and sharp waves or electrographic seizure when presenting with evolution in frequency and amplitude. Although a Moretti et al. study reported overall increase in high Alpha power in temporal brain atrophy, which is contrary to the reported increased Beta power in the current study^75^, the reported Alpha powers were generated from Alpha-3/Alpha-2 ratios, which are basically the results of the so-called Extended Alpha range (5–14 Hz). In this range, Alpha-3 is at the high end of the extended range, which basically represents part of the Beta range in conventional EEG ranges. This published finding indirectly supports the current observation of Beta dominance in brain atrophy.

Results on this work’s analysis of both quantitative EEG markers show a statistically insignificant negative correlation between FOBAR and DAR values in both atrophic cases and normal subjects, which suggest that Beta activities are generated more in chronic brain conditions associated with loss of volume^76^. Delta and Theta activities may be important biomarkers in dysfunctioning acute brain conditions associated with edema^77^. In concurrence with Yoo et al.’s study, this work suggests that, among other indices that can be used for post-stroke evaluation of brain injury, Alpha, Theta, delta, DAR and DTABR are in consideration^41^. Therefore, both this study and certain literature imply that DAR favors acuteness and BAR favors chronicity when encephalopathies are studied with QEEG.

## Conclusion

This study draws three main conclusions. First, the cerebral dysfunctional studies by QEEG may be subject to the etiological nature of brain injuries and chronicity of the disease processes. Second, the Focused Occipital Beta-alpha Ratio (FOBAR) shows significant prospect as a reliable biomarker for studying atrophic encephalopathy. Third, the Delta-Alpha Ratio (DAR) of the Quantitative EEG (QEEG) may be more useful in studies of acute encephalopathy with concurrence of cerebral edema and likewise in space occupying lesions.

## Recommendations

This work recommends that the FOBAR biomarker is studied with a much broad population than the one included herein and presented with upper and lower limits of values among normal subjects as this study’s normal controls accounted for only one-quarter of the sample size. Hence, dedicated research that seeks more accuracy in normal limits of BAR values among subjects with normal brain volume in neuro imaging would add value to the future utility of the newly designed biomarker.

## What is already known

Cerebral dysfunctions are associated with high Delta-alpha ratio because of an increase in slowing waves in theta and delta frequencies. Also, epileptiform discharges happen in both epileptic and non-epileptic patients, and these are important when there is a clinical history of convulsions. Their mechanism of formation is still unknown.

## What is added from this study

In pediatric brain atrophy, the chronicity of the disease process means that focal slowing EEG is not a common finding. Epileptiform discharges are of varying patterns and predominantly generated in children with brain atrophy rather than children with a normal brain. Electrographic seizure as well as polyspike-sharp waves is epileptic patterns profoundly seen in atrophic brains running in beta frequency range.
